# Peptide Nucleic Acid Based Molecular Authentication for Identification of Four Medicinal *Paeonia* Species Using Melting Array Analysis of the Internal Transcribed Spacer 2 Region

**DOI:** 10.3390/molecules22111922

**Published:** 2017-11-07

**Authors:** Wook Jin Kim, Sungyu Yang, Goya Choi, Byeong Cheol Moon

**Affiliations:** K-herb Research Center, Korea Institute of Oriental Medicine, 1672 Yuseong-daero, Yuseong-gu, Daejeon 305-811, Korea; ukgene@kiom.re.kr (W.J.K.); sgyang81@kiom.re.kr (S.Y.); serparas@kiom.re.kr (G.C.)

**Keywords:** *Paeonia* species, peptide nucleic acid (PNA), melting curve analysis, internal transcribed spacer (ITS), Paeoniae Radix

## Abstract

Accurate taxonomic identification of plant materials in herbal medicines is important for product quality control. The genus *Paeonia* (Saxifragales) is the source of the herbal preparations Paeoniae Radix (Paeoniae Radix Alba and Paeoniae Radix Rubra) and Moutan Radicis Cotex. However, confusion has arisen regarding their contents due to linguistic and taxonomic ambiguities, similar morphologies and different definitions of Paeoniae Radix in the Korean and Chinese national pharmacopoeias, leading to the distribution of adulterated products. To develop a method for identifying the four *Paeonia* species used in these medicines, three fluorescently-labeled peptide nucleic acid (PNA) probes were designed against ITS2 sequences containing single nucleotide polymorphisms (SNPs) and used in a real-time PCR melting curve assay. Each of the four *Paeonia* species was accurately identified using this analysis. The accuracy and analytical stability of the PNA melting curve assay was confirmed using commercially available samples of the four *Paeonia* species. This assay is a reliable genetic tool to distinguish between different *Paeonia*-derived herbal medicines and identify the botanical origins of Paeoniae Radix and Moutan Radicis Cortex. This technique may also contribute to quality control and standardization of herbal medicines by providing a reliable authentication tool and preventing the distribution of inauthentic adulterants.

## 1. Introduction

Herbal medicine derived from plants has been used in traditional medicine for conditions including injury and acute pain for more than one thousand years [1,2]. In oriental medicine, one or more species having equal effects can be used to treat the same disease, leading to plant species with different taxonomic classifications having the same herbal medicinal name. Medicinal plants used as sources of oriental medicines and folk remedies are under evaluation to verify their pharmacological efficacy [3], and several studies recently reported pharmacological effects associated with plant species used in traditional medicine [4,5]. To standardize the prescription of herbal medicines, only authentic herbal materials defined in national pharmacopoeias should be used for therapeutic purposes. However, definitions of botanical origin for numerous herbal remedies differ between pharmacopoeias [6], and some inauthentic species are sometimes included. In a similar way, the use of synonyms and homonyms has led to the inclusion of inauthentic substitutions or adulterants in traditional herbal medicines [7]. These inauthentic substitutions or adulterants, which have different pharmacological efficacy or may have toxic components in some cases, are not an appropriate use as herbal material. Therefore, authentic herbal materials should be used to standardize the quality of herbal medicines.

In Korean traditional herbal medicine, *P. lactiflora*, *P. japonica* and *P. veitchii* are considered as Paeoniae Radix, and *P. suffruticosa* is considered as Moutan Radicis Cortex [8]. These herbal medicines are prescribed to treat different symptoms [6,8]. Moutan Radicis Cortex, which is prescribed to activate blood and to treat extravasated blood in the heart, liver and kidney, is defined exclusively as the root bark of *P. suffruticosa* [6,9]. By contrast, Paeoniae Radix, which is used to treat extravasated blood, relieve pain, alleviate fever and induce hepatoprotection, has different botanical origins in the Korean, Chinese and Japanese pharmacopoeias [6,10]. Two different herbal medicines, Paeoniae Radix Rubra (synonymous with Paeoniae Radix) and Paeoniae Radix Alba, are used in Chinese traditional medicine [6]. Paeoniae Radix Rubra is defined as *P. lactiflora* and *P. veitchii*, and Paeoniae Radix Alba has excellent pharmacological effects on anti-inflammatory and immune regulation and is defined exclusively as the peeled root of *P. lactiflora*.

The pronunciation of Paeoniae Radix Alba is similar to that of *P. japonica*, termed Baekjak in Korean, which can cause confusion. *P. japonica* is considered to be more effective than *P. lactiflora* and *P. veitchii* and is more expensive in the Korean herbal market. *P. lactiflora* and *P. veitchii* are distributed as Paeoniae Radix in Korea, and the majority of *P. lactiflora* and *P. veitchii* is imported from China. Peeled bark and sliced roots of *P. lactiflora* are morphologically similar to those of *P. japonica* and are sometimes falsely distributed as *P. japonica* at the farm level and in herbal markets.

Molecular genetic tools such as DNA barcoding can be used to identify the botanical origins of herbal medicines and have a range of advantages such as analytical reliability, cost effectiveness and reproducibility [11,12,13]. Another advantage of DNA-based approaches is that they do not require years of taxonomic training as for plant taxonomist for morphologically accurate identification. DNA barcoding based on short nuclear or organellar DNA sequences is used for the identification of a broad spectrum of organisms including plants, animals, microorganisms and fungi [14,15,16,17]. Several genetic markers, including *rbc*L, *mat*K, *psb*A-*trn*H, *trn*L-F and *rps*16 in the chloroplast genome and ribosomal DNA internal transcribed spacers (rDNA-ITS) in the nuclear genome, are widely used as representative DNA barcode regions for identification of plant species and/or botanical origins of herbaceous medicinal materials [17,18,19]. Although the Plant Working Group of the Consortium for the Barcode of Life (CBOL) recommended the combined use of *mat*K and *rbc*L for plant classification, combined ITS (ITS1 + ITS2) regions or the ITS2 region alone are frequently used for examination of closely related species as the ITS subregions have higher substitution rates than the genetic markers *rbc*L, *mat*K and *rps*16 [18,20]. While DNA barcoding provides accurate species identification, the process is relatively time consuming, and complicated, including processes such as DNA amplification, gel electrophoresis, rescue of amplicon, sub-cloning into T-vector, *Escherichia coli* transformation, sequencing and sequence analysis [7,20]. In addition, the limitations of DNA barcoding using the universal primers are that they are not amplified in some plant taxa [15,21].

Real-time PCR (RT-PCR) is a powerful method that identifies specific amplified fragments during PCR, removing the need for subsequent gel electrophoresis [22,23]. Fluorescent tagging of probes allows discrimination of different products and facilitates multiplexing within a single reaction [24,25]. Melting curve analysis allows the detection of multiple targeted DNA sequences with different melting temperature ™ values in individual fluorescent channels. Melting analysis using peptide nucleic acid (PNA) probes is a powerful method for the detection of single nucleotide polymorphisms (SNPs). PNAs contain an uncharged peptide backbone that has high chemical and thermal stability. Thermal stability differences between perfectly matched and mismatched PNA probe-template duplexes are higher than those in DNA probe-template duplexes, and mismatch sensitivity is therefore higher with PNA probes than with DNA probes. The lower melting temperatures exhibited by mismatched PNA probe-template duplexes compared with perfectly matched duplexes can be distinguished at high resolution. PNA fluorescent melting profile analysis can thus be used for efficient detection and identification of taxonomic origins at the species level [26,27,28].

In this study, we established an SNP-based PNA melting array method to discriminate four medically important *Paeonia* species using RT-PCR with specific PNA probes based on ITS2 sequences. This molecular authentication tool successfully identified the four *Paeonia* species with high resolution and stability. This method can be used to distinguish between two herbal medicines, Paeoniae Radix and Moutan Radicis Cortex, and to identify the botanical origins of *Paeonia*-derived herbal materials, including processed forms such as sliced preparations and ground powders.

## 2. Results

### 2.1. Analysis of ITS2 Sequences

**Figure 1:**
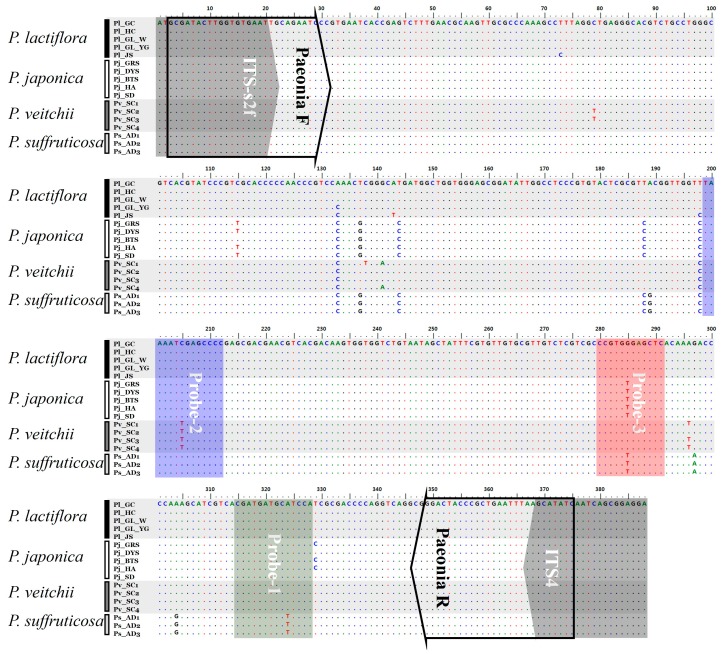
Design of primers and three PNA probes for melting curve analysis. Sequences were designed against ITS2 sequences from *Paeonia* species. Arrows and boxes indicate specific primers used for amplification and PNA probes used for melting curve analysis, respectively.

ITS2 regions were amplified from the 17 *Paeonia* samples listed by Kim et al. [8] using universal primers. PCR amplification efficiency for all four *Paeonia* species was 100%. ITS2 amplicons of one band (approximately 400 bp in length) were separated by agarose gel electrophoresis. PCR products (approximately 400 bp) were sub-cloned into a T-vector, and the inserts were sequenced using T7 and SP6 promoters. We obtained trustworthy sequences from more than three clones considering sequences such as PCR errors, misreading and chimeric and geographic variations and confirmed the species using NCBI BLAST analyses. Full ITS2 sequences were aligned along a length of 388 bases (Table 1 and Figure 1). Intra-species distances for *P. lactiflora*, *P. japonica*, *P. veitchii* and *P. suffruticosa* were 0.47%, 0.26%, 0.61% and 0.00%, respectively (Table 1). Inter-species distances were 1.84%, 1.87%, 2.00% and 2.25%, respectively (Table 1). These results indicated that *P. veitchii* and *P. suffruticosa* had the highest genetic divergences within and between species, respectively. The G + C ratio of ITS2 regions was the highest in *P. suffruticosa* (57.2%), followed by *P. japonica* (56.9%), *P. lactiflora* (56.1%) and *P. veitchii* (55.9%) (Table 1). Species-specific nucleotides (i.e., SNPs) were located from ITS2 sequences at one position in *P. veitchii* and four positions in *P. suffruticosa*, but none were located in *P. lactiflora* and *P. japonica* (Table 3). These results demonstrated that the species-specific nucleotides located from comparative analysis of ITS2 sequences could not be used for identification of all four *Paeonia* species. The ITS2 region was thus unsuitable for standard DNA barcoding in *Paeonia*. Instead, PNA-based probes based on divergent regions of ITS2 were developed for identification of the four *Paeonia* species. In previous study, we reported that ITS1 regions have more variable sequences and species-specific nucleotides compared to ITS2, but ITS1 was inappropriate to design PNA probes for identifying *Paeonia* species because of its sequence variability [8].

### 2.2. Establishment of a PNA Melting Array Method for Identification of Paeonia Species

**Figure 2:**
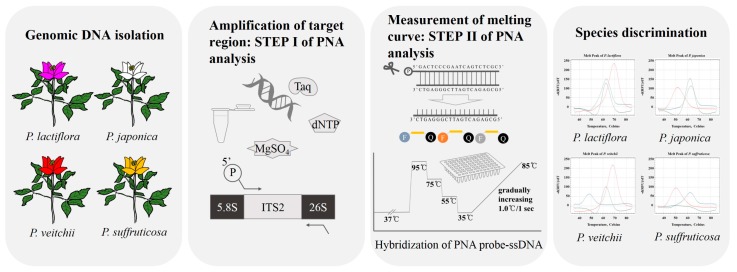
RT-PCR amplification and PNA probe melting curve analysis of four *Paeonia* species. A region of the ITS2 sequence was amplified, and melting analysis with three fluorescently-labeled PNA probes was used to discriminate between the four species.

The melting array analysis method using RT PCR and PNA probes is illustrated in Figure 2. *Paeonia* F and R primers were designed to amplify a 373-bp internal ITS2 region in all four *Paeonia* species (Table 2 and Figure 1). Abundant amplicons were produced that were suitable for melting array analysis (Supplemental Figure S1). To confirm amplicon identity, PCR products were sequenced (Sanger method) and compared to known ITS2 sequences from the four *Paeonia* species using blastn of NCBI Standard Nucleotide BLAST. The amplified sequences were identical to the corresponding ITS2 database sequences and previously obtained ITS2 sequences, demonstrating that the *Paeonia* F and R primers amplified a unique target region in all four *Paeonia* species.

**Figure 3:**
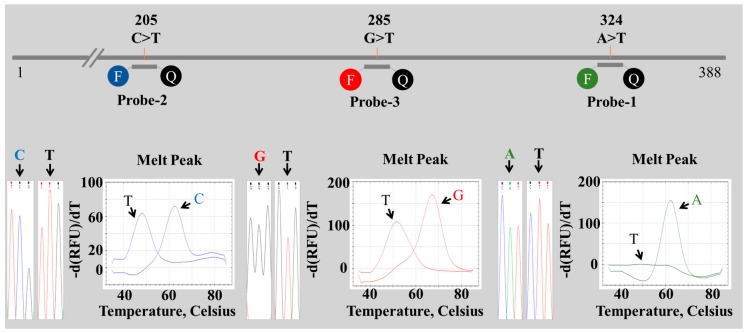
Melting curve analysis of perfectly matching and mismatching duplexes between PNA probes and amplified ITS2 sequences. PNA probes were dually labelled with HEX (Probe 1), FAM (Probe 2) or Texas Red (Probe 3) and a quencher at opposite ends.

Multiple alignment sequence analysis located nine candidate regions for probe design, with four polymorphic nucleotides at positions 137 (T/G), 144 (T/C), 188 (T/C) and 285 (G/T) and five species-specific nucleotides at positions 189 (G in *P. suffruticosa*), 205 (T in *P. veitchii*), 297 (A in *P. suffruticosa*), 304 (G in *P. suffruticosa*) and 324 (T in *P. suffruticosa*) (Figure 1 and Table 3). Regions corresponding to three of these nucleotide positions, 205, 285 and 324 (Figure 1 and Table 2), were used for PNA probe design. Alignment of the PNA probes (dually labeled with HEX, FAM or Texas Red and a quencher at opposite ends) to template is illustrated in Figure 1 and Figure 3. Probe regions exhibited no intra-species variation, and three probes was needed for *Paeonia* species discrimination (Figure 1 and Table 2).

**Figure 4:**
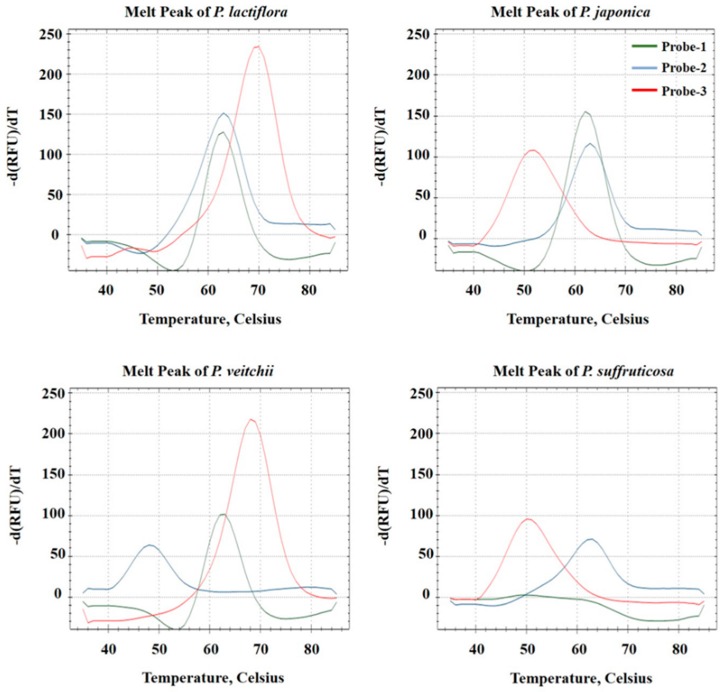
Melting curve profiles of four *Paeonia* species with three ITS2 PNA probes. Perfectly matched probes produced melting peaks at 63 °C (Probe 1; green), 63 °C (Probe 2; blue) and 70 °C (Probe 3; red). Corresponding mismatches produced no curve (Probe 1), 48 °C melting peak (Probe 2) or 52 °C melting peak (Probe 3).

Species-specific nucleotides at positions 324 (A/T) and 205 (C/T) were used to design Probes 1 and 2, respectively (Figure 3). Probe 1 (labeled with HEX) hybridized perfectly with *P. lactiflora* (A), *P. japonica* (A) and *P. veitchii* (A), but not with *P. suffruticosa* (T). *P. lactiflora*, *P. japonica* and *P. veitchii* formed a melting peak at 63 °C with Probe 1, whereas no melting peak within the 35–85 °C range was seen with *P. suffruticosa* (Figure 3). Probe 2 (labeled with FAM) hybridized perfectly with *P. lactiflora* (C), *P. japonica* (C) and *P. suffruticosa* (C), but not with *P. veitchii* (T). *P. veitchii* formed a melting peak at 48 °C with Probe 2, and the other three *Paeonia* species exhibited a peak at 63 °C (Figure 3). Probe 3 (labeled with Texas Red) hybridized perfectly with *P. japonica* (G) and *P. suffruticosa* (G) and formed a mismatch hybrid with *P. lactiflora* (T) and *P. veitchii* (T). *P. lactiflora* and *P. veitchii* formed a melting peak at 70 °C with Probe 3, and *P. japonica* and *P. suffruticosa* formed a melting peak at 52 °C (Figure 3). Each of the three probes was detected separately using distinct fluorescence channels. Melting analysis was performed with 1 °C increments over the 35–85 °C range to produce melting curve profiles (Figure 4). No adverse results were observed with a no template control (NTC), and no fluorescent signal was observed from the probe dimer of non-specific products. In addition, to confirm the duplexes of the PNA probe-template, we analyzed the hybridization of the PNA probe and template DNA using the PNA probe, the PNA probe and template mixture and the renatured PNA probe-template on gel electrophoresis. As shown in Supplemental Figure S2, Lane 3, PNA probe-template duplexes were observed with 373-bp templates at approximately 850 bp on agarose gel electrophoresis.

Melting curve profiles were scored for perfect matches (1) or mismatches (0), and each of the four *Paeonia* species was represented by a unique barcode: *P. lactiflora*, 111; *P. japonica*, 110; *P. veitchii*, 101; and *P. suffruticosa*, 010 (Table 4).

### 2.3. Discrimination of Four Paeonia Species Based on Melting Array Analysis

**Figure 5:**
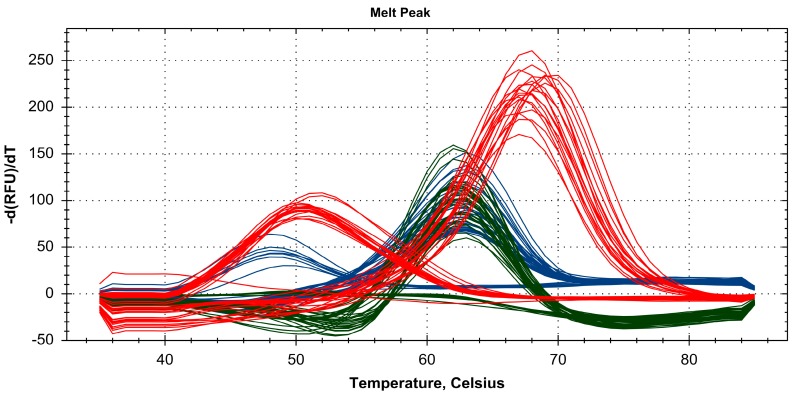
Melting profiles of *Paeonia* samples cultivated in Korea and China derived from melting curve analysis with PNA probes. Green, blue, and red indicates the melting peaks generated by HEX (Probe 1), FAM (Probe 2), and Texas Red (Probe 3), respectively.

To test the robustness of the RT-PCR–PNA probe melting analysis identification method, 21 additional *Paeonia* samples were collected from diverse farmed locations in Korea and China and assessed. Six, seven, three and five samples of *P. lactiflora*, *P. japonica*, *P. veitchii* and *P. suffruticosa* were obtained, respectively. Figure 5 shows the merged melting curve profiles of 21 commercial *Paeonia* samples using the three PNA probes. As shown in Table 5, all 21 samples were successfully discriminated, and only one sample was inauthentic (Table 5 and Figure 5). This sample was described as *P. japonica*, but was identified as *P. lactiflora*. To confirm the accuracy of RT-PCR-PNA probe melting analysis, we analyzed nucleotide sequences of those PCR products using 373-bp amplicons of 21 samples, which were amplified using the *Paeonia* F and R primers (Supplemental Figure S3A). The resulting sequences were identical to the corresponding ITS2 sequences of the four *Paeonia* species excluding one sample, Sample No. 9 (Supplemental Figure S3B). This sample was identified as *P. japonica* in morphology-based species identification, but it was identified and confirmed as *P. lactiflora* in RT-PCR-PNA probe melting analysis.

## 3. Discussion

The same herbal medicine is often associated with different plant species in different national pharmacopoeias, which sometimes leads to adulteration and misidentification of herbal remedies [6,7]. Identifying the botanical origins of herbal medicines can be challenging, particularly where herbal medicines are distributed in highly processed forms such as powders or slices [7,14]. Morphological and genetic features are the main characteristics used for discrimination of herbal medicine species [11,14,20]. Morphological identification of processed herbal medicines requires professional expertise and taxonomic keys that accurately identify features such as color, size and shape of the sliced and whole plant parts [29]. DNA barcodes and molecular marker-based genetic traits are used for accurate identification of herbal medicines [12,20]. In contrast to molecular markers such as simple sequence repeats (SSRs) or random amplified polymorphic DNAs (RAPDs), which do not usually have broad utility, DNA barcoding uses short DNA regions that are found in diverse organisms. In plants, common sequences located in the nuclear and organellar (chloroplast, mitochondria) genomes are widely used for barcoding [20].

Several DNA barcode regions were investigated previously for their efficiency in identifying original herbal medicine species and closely related adulterants. Chen et al. [21] evaluated the utility of several plant barcoding sequences (ITS, ITS2, *mat*K, *rbc*L, *psb*A-*trn*H, *rpo*C1 and *ycf*5) for identifying more than 6600 medicinal plants and their closely related species and proposed ITS2, with a success rate of 92.7%, as the most suitable barcoding sequence.

Previously, we evaluated DNA barcoding of four *Paeonia* species with ITS, *mat*K and *rbc*L sequence regions [8] and found that ITS and *rbc*L were both discriminatory at the species level. However, DNA barcoding is something of a cumbersome process involving PCR amplification, sub-cloning, sequencing and data analysis, and we wished to develop a simple genetic technique for discrimination of the four *Paeonia* species [30]. The sequence-characterized amplified region (SCAR) marker has been known as one of the accurate and simple molecular markers for species identification in medicinal plants, and SCAR markers based on universal DNA barcode regions were developed for several medicinal plants [7,20,30]. An ideal SCAR marker based on DNA barcoding region contains (1) short sequences in a 1-kb region that can be routinely amplified by PCR, (2) species-specific nucleotides distinguishable among the allied species and (3) minimal intra-species variation [20,30]. However, these regions (ITS, *mat*K and *rbc*L) did not harbor sufficient species-specific nucleotides to allow SCAR marker development for *Paeonia* species [8]. We therefore used an RT PCR approach with PNA probe melting curve analysis to develop a multiplex-discriminable marker for the four *Paeonia* species.

Several genotyping applications using melting curve analysis were reported previously in plants [31,32,33]. Melting curve analysis using PNA probes, which are artificially synthesized DNA analogs with an uncharged peptide backbone, can be combined with various fluorescent dye to allow multiplex discrimination of DNA variants with insufficient species-specific marker nucleotides [26]. This method exploits differences in melting temperatures between perfectly matched and mismatched DNA-PNA probe duplexes. Here, the ITS2 region was selected for melting curve analysis of *Paeonia* species. ITS2 had sufficient inter-species sequence variation to allow PNA probe design, had minimal intra-species variation and was more amenable to PCR amplification than ITS and *rbc*L, with 100% PCR efficiency. In this study, a region of the ITS2 sequence was amplified, and melting curve analysis was performed using three PNA probes labeled with distinct fluorescent tags. This technique allowed discrimination of four *Paeonia* species in a single reaction. The PNA probe-based method established in this study is a rapid and sensitive method for discriminating *Paeonia* species.

PNA probe techniques that utilize the strong DNA-binding capacity of PNA can be used to discriminate herbal medicine species from allied species and from morphologically similar adulterant species in herbal preparations. Abundant amplicons are produced by the RT-PCR step of this technique, allowing discrimination of small amounts of material in mixed preparations without the need for further analysis steps such as gel electrophoresis. Melting curve analysis of the four *Paeonia* species offers substantial advantages similar to DNA barcoding analysis such as ITS and *mat*K, including fast and accurate species discrimination. The mixed adulterants could be distributed in herbal markets because herbal medicines are usually distributed as processed forms (powders or slices). Therefore, the discrimination of botanical origin is very important for quality control of herbal medicines. Melting curve analysis could be applied to discriminate the mixture of two *Paeonia* species using the three PNA-probes. In theory, the multiple mixtures excluding *P. suffruticosa* also could discriminate the botanical origins depending on the meting temperature (*T*_m_) and fluorescence intensity (height of melting peak) of individual PNA probes. For example, the mixture of three *Paeonia* species, *P. lactiflora*, *P. japonica* and *P. veitchii*, would be detected at *T*_m_ 63 °C with Probe 1, at both 52 °C and 63 °C with Probe 2 and at both 52 °C and 70 °C with Probe 3, respectively. However, the fluorescence intensity of Probes 2 and 3 would be detected half against perfect match (PM) at the *T*_m_ of mismatch (MM). In contrast, two species mixtures between *P. lactiflora*, *P. japonica* and *P. veitchii*, would be detected with the same intensity of fluorescence at both MM and PM. These results indicate that an additional PNA probe specific to *P. lactiflora* or *P. japonica* is needed to establish an ideal method for discriminating the botanical origins of three or four *Paeonia* species mixtures. However, we could not find appropriate PNA probe regions in the nrDNA-ITS region, as well as *mat*K and *rbc*L genes for four *Paeonia* species [8].

The melting curve analysis method established in this study was used to test 21 commercial samples of four *Paeonia* species, *P. lactiflora*, *P. japonica*, *P. veitchii* and *P. suffruticosa*, obtained from farms in Korea and China. We could not detect mixed adulterants in 21 commercial medicinal samples analyzed in this study. Of these samples, one *P. japonica* sample was revealed to be *P. lactiflora*. *P. japonica* is of higher value than other *Paeonia* species in the Korean herbal market, and adulteration of *P. japonica* preparations with other *Paeonia* species is possible. The accurate melting curve technique developed in this study allows the accurate discrimination of four *Paeonia* species and will contribute to quality control and standardization of Paeoniae Radix and Moutan Radicis Cortex.

## 4. Materials and Methods

### 4.1. Plant Materials and DNA Extraction

Seventeen *Paeonia* samples from four species, *P. lactiflora*, *P. japonica*, *P. veitchii* and *P. suffruticosa*, were used to establish a PNA-based melting array analysis method. All samples were collected from native habitats or farming fields in Korea and China, and species were identified based on analysis of morphological features by the Classification and Identification Committee of the Korea Institute of Oriental Medicine (KIOM), which comprises experts in the fields of plant taxonomy, botany, ecology, pharmacognosy and herbology. All plant materials were prepared voucher specimens that were preserved in the herbarium of the KIOM (Index Herbariorum code: KIOM) (Supplemental Photo 1). Fresh leaves of samples were quick frozen in liquid nitrogen and stored at −80 °C. To validate the PNA-based melting array method of distinguishing between *Paeonia* species, 21 commercial medicinal samples were purchased from herbal markets in Korea and China and tested.

Approximately 100 mg of frozen leaves were crushed in 800 μL AP1 buffer (DNeasy^®^ Plant Mini Kit, Qiagen, Valencia, CA, USA) using a Precellys™ Grinder (Bertin Technologies, Montigny-le-Bretonneux, France). Genomic DNA was extracted using the DNeasy^®^ Plant Mini Kit according to the manufacturer’s protocol. Genomic DNA quality was confirmed using 1.5% agarose gel electrophoresis with a 1-kb DNA ladder (Solgent, Daejeon, Korea), and DNA concentration was determined with UV/Vis spectrophotometry by absorbance at 260 nm (ND-1000, NanoDrop, Wilmington, DE, USA). DNA was diluted to a final concentration of ~15 ng/μL and used as a template for PCR amplification.

### 4.2. Amplification and Sequencing of ITS2 Regions

ITS2 regions were amplified from 17 *Paeonia* samples (*P. lactiflora*, *P. japonica*, *P. veitchii* and *P. suffruticosa*) using ITS-s2f and ITS4 universal primers (0.5 µM of each primers). PCR conditions were as described in Table 2. PCR amplification was performed using Solg™ 2 × Taq PCR Smart-Mix I (Solgent). The amplified products were separated using 1.5% agarose gel electrophoresis with a 100-bp DNA ladder (Solgent, Daejeon, Korea). DNA fragments were excised from the gel and extracted using a Gel Extraction Kit (Qiagen, Valencia, CA, USA). Eluted DNA fragments were sub-cloned into pGEM^®^-T Easy Vector (Promega, Madison, WI, USA) and transformed into *E. coli* using a JUMBO-80 kit with HIT-JM109 (RBC Bioscience, Taipei, Taiwan) competent cells according to the manufacturers’ protocols. Transformed *E. coli* were spread on LB agar medium containing 100 μg/mL ampicillin, 40 μg/mL X-gal and 0.5 mM IPTG and incubated for 18–20 h at 37 °C. Plasmid DNA was isolated from white colonies, and insert sequences were determined using dideoxynucleotide chain termination sequencing (Sanger sequencing) with standard SP6 and T7 primers. Raw ITS2 sequences were obtained from a minimum of three bacterial colonies for each of the 17 *Paeonia* samples. Nilsson et al. [34] suggested guidelines for quality control of barcoding sequence. Thus, we edited raw ITS2 sequences considering on target genes, orientation of sequences and chimeras and also confirmed species using NCBI BLAST. Sequences were assembled using the BioEdit Version 7.2.5 software, and the representative sequences of individual samples were determined [35]. These representative ITS2 sequences of individual samples were registered in the NCBI GenBank (*P. lactiflora*, MG210828-MG210832; *P. japonica*, MG210833-MG210837; *P. veitchii*, MG210838-MG210841; *P. suffruticosa*, MG210842-MG210843). These individual ITS2 sequences were aligned to analyze the intra-/inter-species genetic distance using the Kimura-2-parameter (K2P) model in the MEGA6 (Molecular Evolutionary Genetics Analysis Version 6.0, Tempe, AZ, USA) program.

### 4.3. Comparative Analysis of ITS2 Sequences for the Design of PNA Probes

Candidate ITS2 sequence regions that included species-specific variable sequences were identified by comparative analysis and used to design PCR primers (*Paeonia* F and R) and PNA probes for the melting curve analysis method. Three specific PNA probes dually labeled with an HEX, FAM or Texas Red fluorophore and a quenchers (dabcyl) at opposite ends were synthesized commercially (SeaSun Biomaterials, Daejeon, Korea). The probes are designed to form a hairpin structure that places the fluorescence and quencher in close proximity to maximize the clamping effect. Probes were purified using high-performance liquid chromatography, and purity was confirmed by mass spectrometry. Probe matching capacities were confirmed by test hybridizations between PNA and complementary target DNA using a CFX96™ Real-Time System (C1000™ Thermal Cycler, Bio-Rad, Hercules, CA, USA).

### 4.4. Establishment of PNA-Based Melting Array Method

The target ITS2 region for hybridization to PNA probes was amplified using 5’-phosphorylated *Paeonia* F primer (0.5 µM), *Paeonia* R primer (0.5 µM) and 2 × q-PCR Pre-mix™ (SeaSun Biomaterials, Daejeon, Korea), as described in Table 2. Specificity and sequence of PCR products were confirmed by 1.5% agarose gel electrophoresis and Sanger sequencing, respectively. After PCR amplification, 9 μL amplicon containing phosphate groups at the 5’ end of all antisense sequences were used for the melting curve analysis. The hybridization between PNA probe and amplicon was reacted using 2× melting array Buffer A, melting array Buffer B and 0.5 µM of each PNA probe, as described in Table 2. PCR amplification of the target region and hybridization between probes and amplicon were performed using a CFX96™ Real-Time System (C1000™ Thermal Cycler, Bio-Rad, Hercules, CA, USA). As described in Table 2, Step 1 was carried out at 37 °C for 1 h to digest the 5’-phosphorylated strands of PCR amplicons by 5’→3’ exonuclease activity. Then, Step 2 was conducted to anneal PNA probes and templates at 75 °C for 1 min, 55 °C for 1 min and 35 °C for 3 min after denaturation at 95° for 2 min. Finally, PNA probe-template duplexes were gradually dissociated from 35 °C to 85 °C with increasing by 1 °C/s in Step 3. The fluorescence of PNA probe dissociated with template was detected in three separate fluorescent channels (HEX, FAM and Texas Red), and melting profiles were analyzed using Bio-Rad CFX Manager Version 3.1 (Bio-Rad, Hercules, CA, USA). The hybridization of PNA probe-template was confirmed on gel electrophoresis using renatured samples (Supplemental Figure S2). Perfectly matched PNA probe-template duplexes were dissociated at a higher temperature than imperfectly hybridized duplexes because of the difference in DNA-binding capacity. The melting temperature differences between perfectly matching and mismatching duplexes were used to score melting curves as 1 or 0 for perfectly matching and mismatching duplexes, respectively. The score combinations for the three probes allowed the four *Paeonia* species to be identified with high accuracy.

## 5. Conclusions

ITS2 sequences of four *Paeonia* species that are used as Paeoniae Radix (*P. lactiflora*, *P. japonica* and *P. veitchii*) or Moutan Radicis Cortex (*P. suffruticosa*) in traditional Korean herbal medicine were analyzed by ClustalW multiple alignment to identify polymorphic nucleotides. PNA probes (dually labelled with an HEX, FAM or Texas Red fluorophore and a quencher at opposite ends) were designed against regions containing polymorphic nucleotides and used to develop a melting curve method for the discrimination of four *Paeonia* species. The method was tested using commercially available *Paeonia* samples from Korea and China. Of 21 samples, one was found to be *P. japonica* rather than *P. lactiflora*, indicating the possible adulteration of *P. japonica* preparations. The PNA melting curve method allowed simple, rapid identification of four *Paeonia* species and will be useful for quality control and standardization of *Paeonia* herbal medicines.

## Figures and Tables

**Table 1:** Statistical characteristics of ITS2 regions.

Scientific Name	Amplicon Length (bp)	Intra-Species Distance (%)	Inter-Species Distance (%)	G + C (%)
*P. lactiflora*	388	0.47 ± 0.45	1.84 ± 0.53	56.1
*P. japonica*	388	0.26 ± 0.17	1.87 ± 0.35	56.9
*P. veitchii*	388	0.61 ± 0.32	2.00 ± 0.69	55.9
*P. suffruticosa*	388	0.00 ± 0.00	2.25 ± 0.66	57.2

**Table 2:** Primer sequences and PCR parameters for ITS2 amplification and PNA melting analysis.

Primer Name	Primer Sequence (5′ to 3′)	PCR Parameter	Reaction Component
ITS-s2f ITS4	ATG CGA TAC TTG GTG TGA AT TCC TCC GCT TAT TGA TAT GC	Step 1: 95 °C, 2 min Step 2: 95 °C, 40 s 53 °C, 30 s 72 °C, 40 s (35 cycles) Step 3: 72 °C 5 min	gDNA: 1 µL (15 ng/µL) Primer: each 2 µL (10 pmol/µL) 2 × Pre-mix: 20 µL Final vol.: 40 µL
*Paeonia* F *Paeonia* R	GCG ATA CTT GGT GTG AAT TGC AGA ATC GAT ATG CTT AAA TTC AGC GGG TAG TCC	Step 1: 95 °C, 10 min Step 2: 95 °C, 30 s 60 °C, 40 s 72 °C, 40 s (33 cycles) Step 3: 72 °C, 1 min 20 °C, 1 min	gDNA: 1 µL (15 ng/µL) Primer: mixture 1 µL 2 × qPCR Pre-mix: 10 µL Final vol.: 20 µL
PNA Probe-1 PNA Probe-2 PNA Probe-3	CGA TGA TGC ATC CA TAA ATC GAG CCC CG CCG TGG GAG CTC	Step 1: 37 °C, 1 h Step 2: 95 °C, 2 min 75 °C, 1 min 55 °C, 1 min 35 °C, 3 min Step 3: 35–85 °C, increasing 1 °C/s	Amplicon DNA: 9 µL 2 × melting array Buffer A: 10 µL Melting array Buffer B: 0.5 µL PNA probe mixture: 0.5 µL Final vol.: 20 µL

**Table 3:** Species-specific nucleotides and polymorphic nucleotides in ITS2 sequences from four *Paeonia* species.

Species	Aligned Nucleic Acid Position								
	137	144	188	189	205	285	297	304	324
*P. lactiflora*	T	T	T	A	C	G	G	A	A
*P. japonica*	G	C	C	A	C	T	G	A	A
*P. veitchii*	T	T	T	A	**T**	G	G	A	A
*P. suffruticosa*	G	C	C	**G**	C	T	**A**	**G**	**T**

Underlined bold characters indicate species-specific nucleotides.

**Table 4:** Barcodes of four *Paeonia* species based on melting curve analysis with three PNA probes.

Species	Probe 1			Probe 2			Probe 3			Barcode
	NT ^1^	*T*_m_^2^	MT ^3^	NT ^1^	*T*_m_^2^	MT ^3^	NT ^1^	*T*_m_^2^	MT ^3^	
*P. lactiflora*	A	63 °C	PM	C	63 °C	PM	G	70 °C	PM	1 1 1
*P. japonica*	A	63 °C	PM	C	63 °C	PM	T	52 °C	MM	1 1 0
*P. veitchii*	A	63 °C	PM	T	48 °C	MM	G	70 °C	PM	1 0 1
*P. suffruticosa*	T	ND	MM	C	63 °C	PM	T	52 °C	MM	0 1 0

^1^ NT: nucleotide type; ^2^ *T*_m_: melting temperature; ^3^ MT: matching type: perfect match (PM) or mismatch (MM).

**Table 5:** Discrimination of *Paeonia* samples using PNA melting analysis.

No.	Collection Site	Sample Name	Barcode	Discrimination Result
1	Tongyoung, Gyeongnam, Korea	*P. lactiflora*	1 1 1	*P. lactiflora*
2	Jeongseon, Gangwon, Korea	*P. lactiflora*	1 1 1	*P. lactiflora*
3	Jinju, Gyeongnam, Korea	*P. lactiflora*	1 1 1	*P. lactiflora*
4	Sejong, Sejong, Korea	*P. lactiflora*	1 1 1	*P. lactiflora*
5	Inje, Gangwon, Korea	*P. lactiflora*	1 1 1	*P. lactiflora*
6	Yanji, Jilin, China	*P. lactiflora*	1 1 1	*P. lactiflora*
7	Andong, Gyeongbuk, Korea	*P. japonica*	1 1 0	*P. japonica*
8	Geochang, Gyeongnam, Korea	*P. japonica*	1 1 0	*P. japonica*
9	Sejong, Sejong, Korea	*P. japonica* *	1 1 1	P. lactiflora *
10	Geumsan, Chungnam, Korea	*P. japonica*	1 1 0	*P. japonica*
11	Hamyang, Gyeongnam, Korea	*P. japonica*	1 1 0	*P. japonica*
12	Jeongseon, Gangwon, Korea	*P. japonica*	1 1 1	*P. japonica*
13	Sancheong, Gyeongnam, Korea	*P. japonica*	1 1 1	*P. japonica*
14	Ngawa, Sichuan, China	*P. veitchii*	1 0 1	*P. veitchii*
15	Ngawa, Sichuan, China	*P. veitchii*	1 0 1	*P. veitchii*
16	Gannan, Gansu, China	*P. veitchii*	1 0 1	*P. veitchii*
17	Jinju, Gyeongnam, Korea	*P. suffruticosa*	0 1 0	*P. suffruticosa*
18	Jinju, Gyeongnam, Korea	*P. suffruticosa*	0 1 0	*P. suffruticosa*
19	Jinju, Gyeongnam, Korea	*P. suffruticosa*	0 1 0	*P. suffruticosa*
20	Jinju, Gyeongnam, Korea	*P. suffruticosa*	0 1 0	*P. suffruticosa*
21	Jinju, Gyeongnam, Korea	*P. suffruticosa*	0 1 0	*P. suffruticosa*

Asterisk (*) indicates inauthentic sample.

## Supplementary Materials

Supplementary materials are available online.
