# Supplementary material for: Peptide Nucleic Acid Based Molecular Authentication for Identification of Four Medicinal Paeonia Species Using Melting Array Analysis of the Internal Transcribed Spacer 2 Region
Source: Molecules. 2017 Nov 7;22(11):1922. doi: 10.3390/molecules22111922 (PMC6150393; doi:10.3390/molecules22111922)
Supplement: Supplementary file 1 [file molecules-22-01922-s001.zip › Supplemental data_molecules-232549 final.docx]

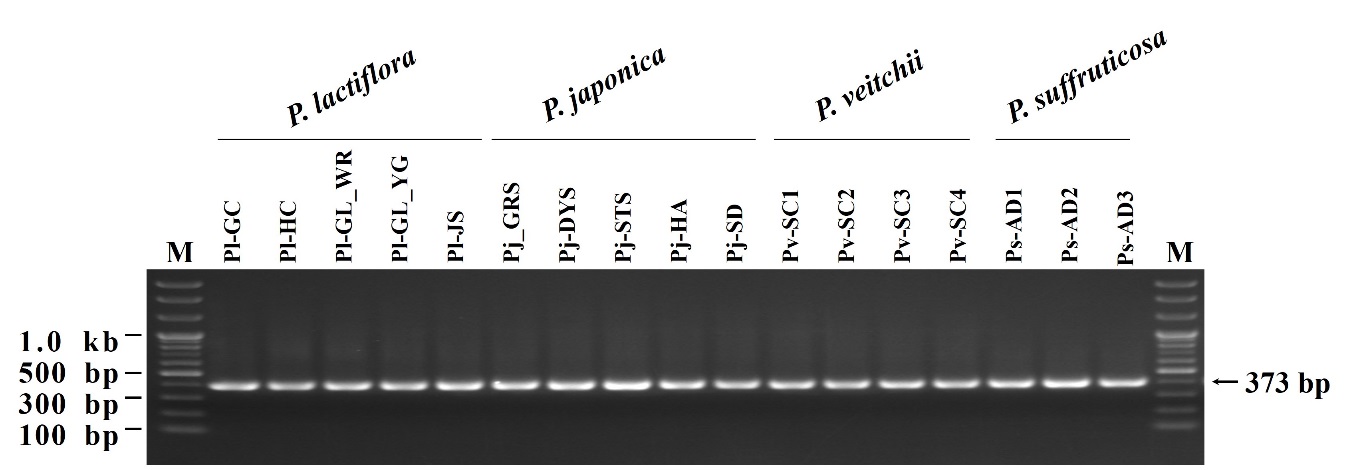


Supplemental Figure 1. PCR amplification of four *Paeonia* species using the Paeonia F/R primer used for RT-PCR–PNA probe melting analysis.


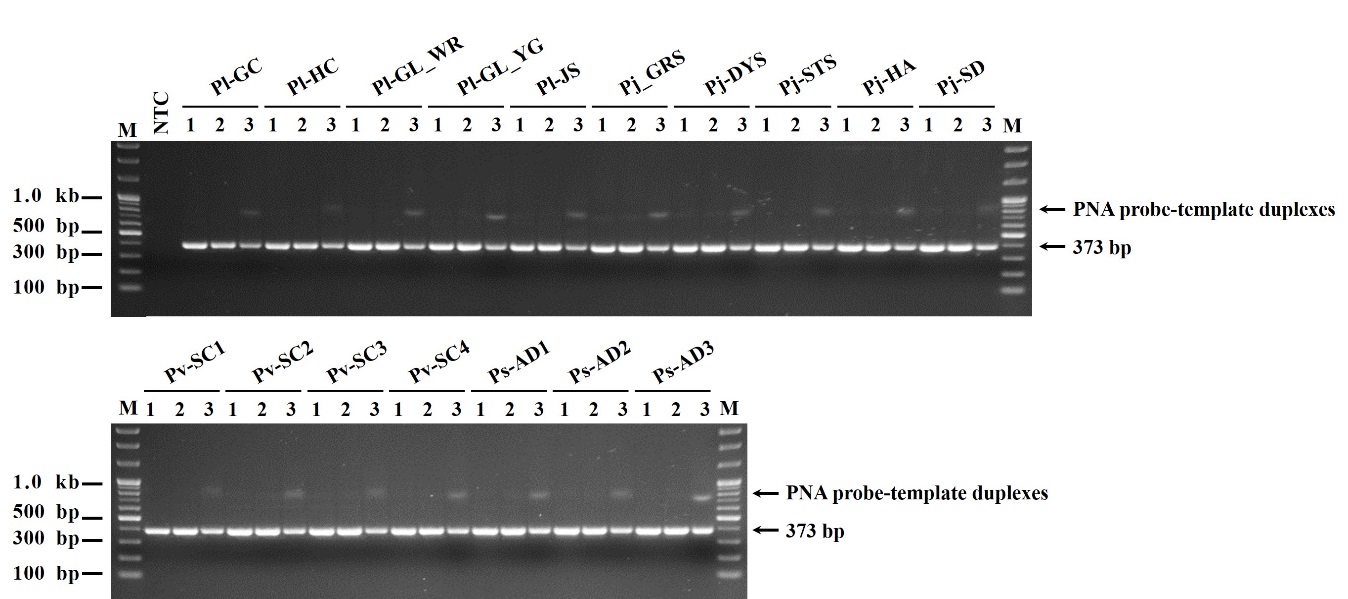


Supplemental Figure 2. Gel electrophoresis for confirmation of the PNA probe-template hybridization. 1: DNA templates; 2: 0.5 µM PNA probe and template mixtures; 3: 0.5 µM PNA probe and template mixtures renatured after melting curve assay; NTC: 0.5 µM PNA probes renatured after melting curve assay with no template.


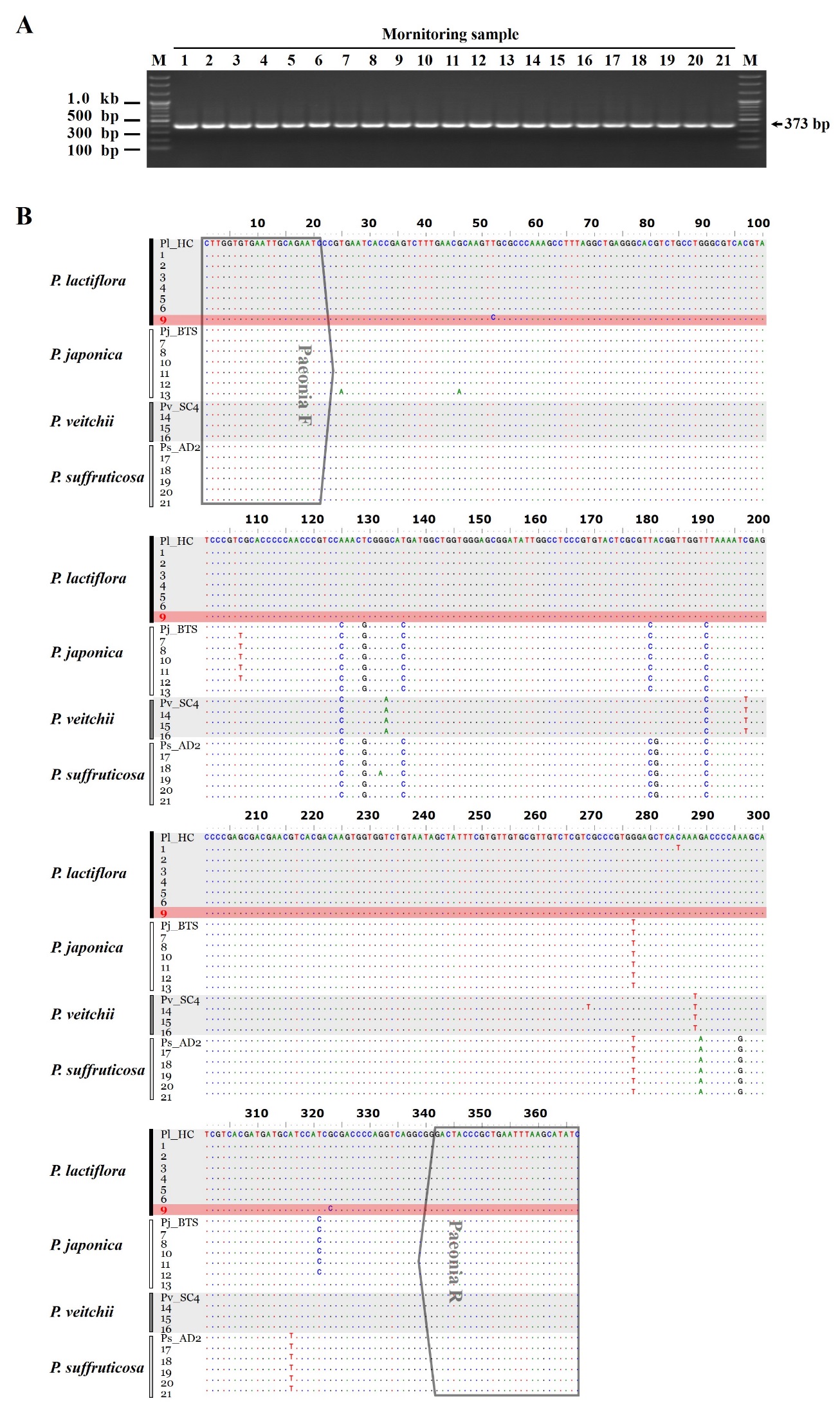


Supplemental Figure 3. Confirmation of PNA melting assay results for 21 *Paeonia* samples. A. Confirmation of PCR amplification for 21 *Paeonia* samples listed in the Table 5. B. Confirmation of sequences of PNA melting assay results amplified by Paeonia F/R primers. Arrows indicate Paeonia F/R primers. Numbers, 1-21, indicate sample no. corresponding to Table 5. The sample sequence colored with orange (sample no. 9) indicates the misidentified sample. Dots (.) indicates the same nucleotide sequences with sample Pl-HC of *P. lactiflora*.


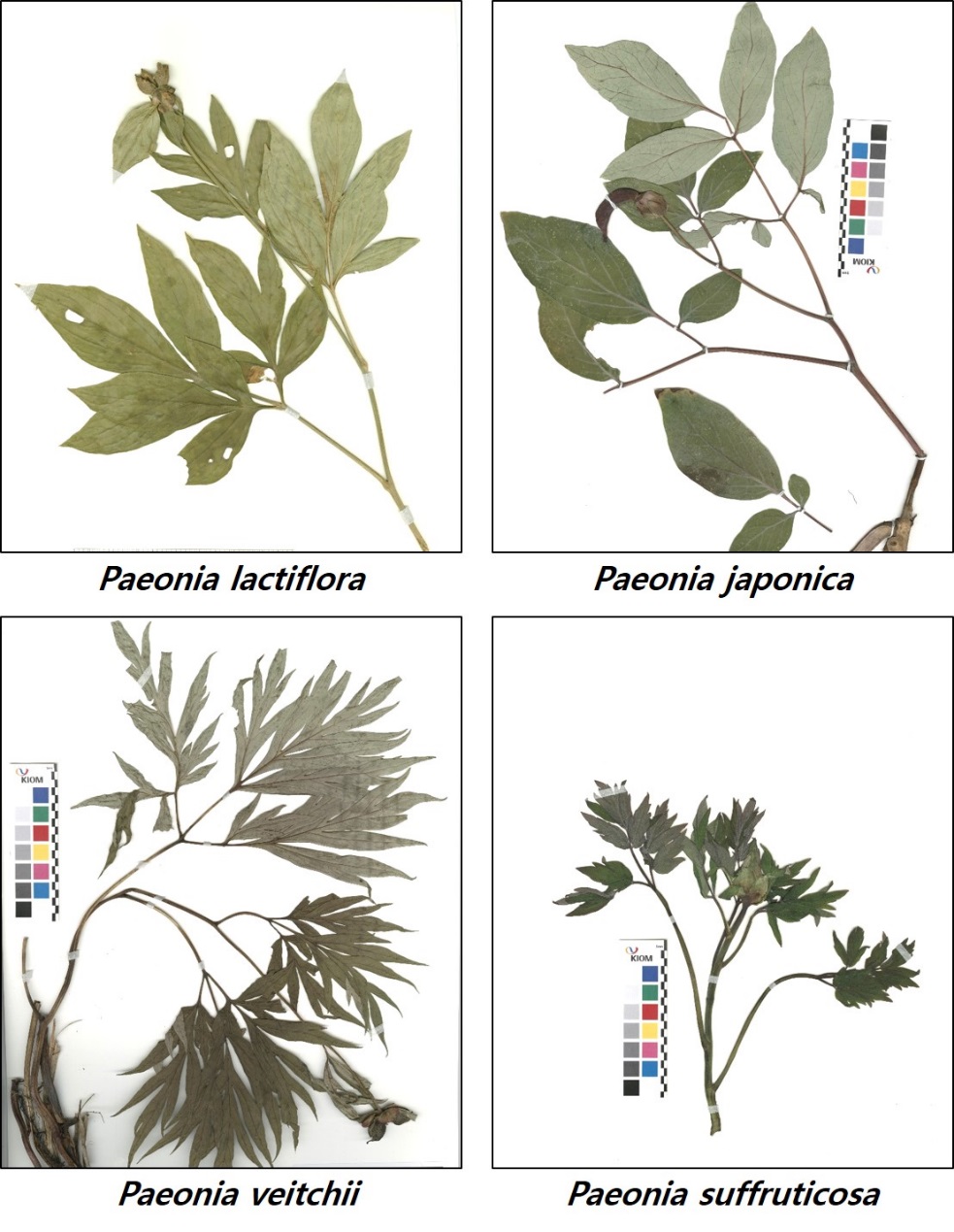


Supplemental Photo 1. Photos of four *Paeonia* species plant specimens used in this study.
