# Supplementary figures and images for: Peptide Nucleic Acid Based Molecular Authentication for Identification of Four Medicinal Paeonia Species Using Melting Array Analysis of the Internal Transcribed Spacer 2 Region
Source: Molecules. 2017 Nov 7;22(11):1922. doi: 10.3390/molecules22111922 (PMC6150393; doi:10.3390/molecules22111922)

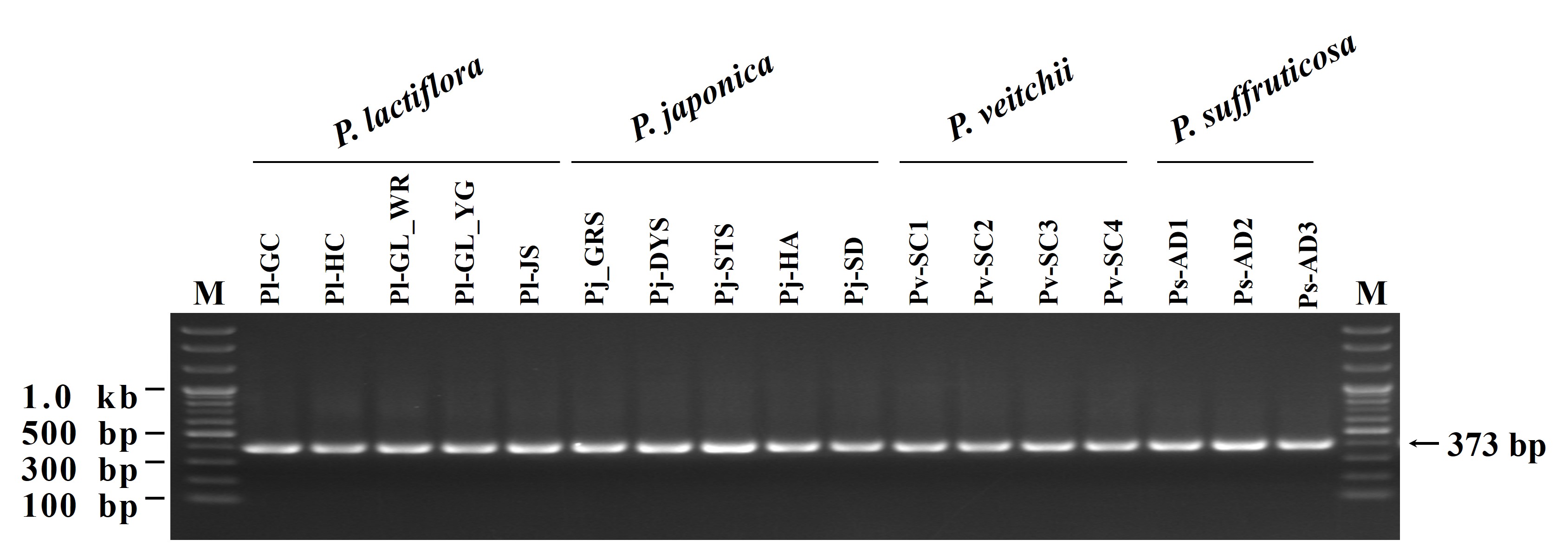

Supplement: Supplementary file 1 [file molecules-22-01922-s001.zip › Supplemental Figure 1..jpg]

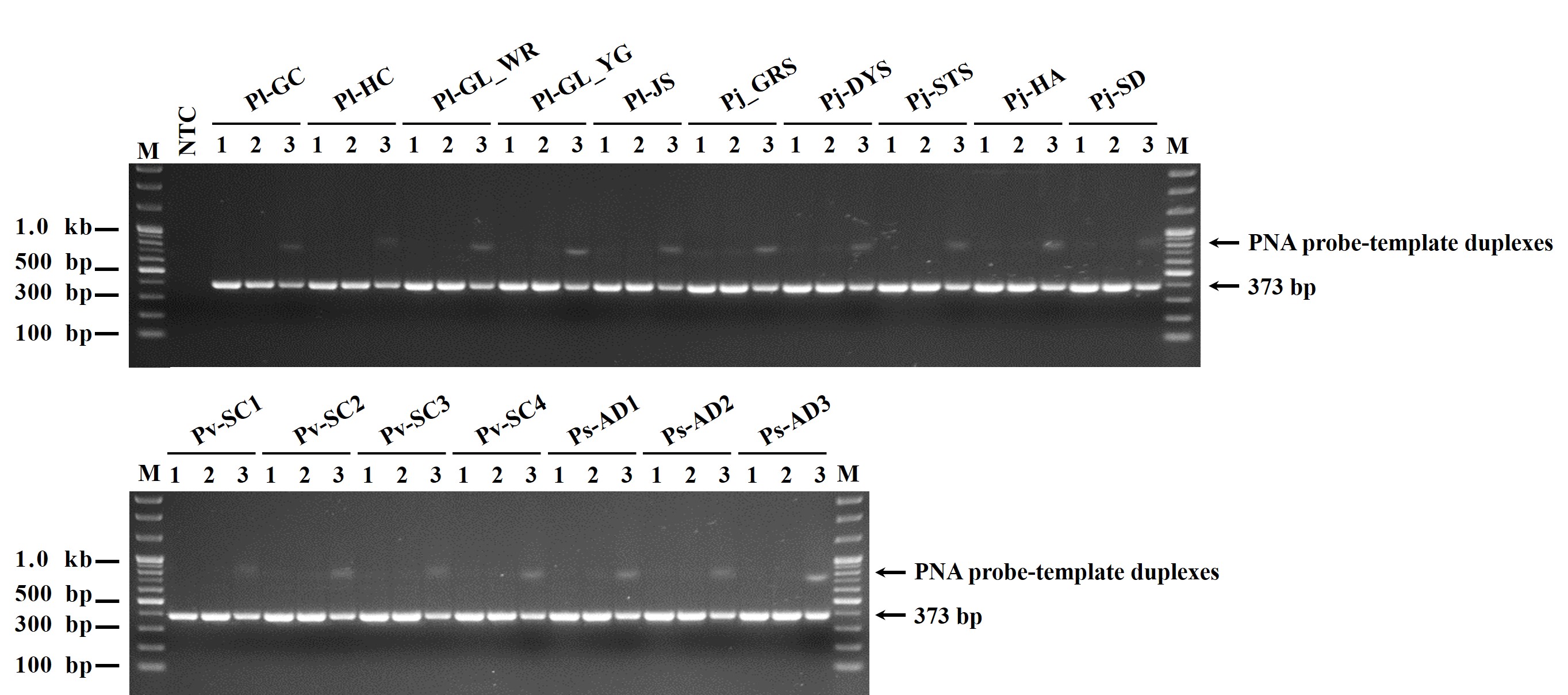

Supplement: Supplementary file 1 [file molecules-22-01922-s001.zip › Supplemental Figure 2..jpg]

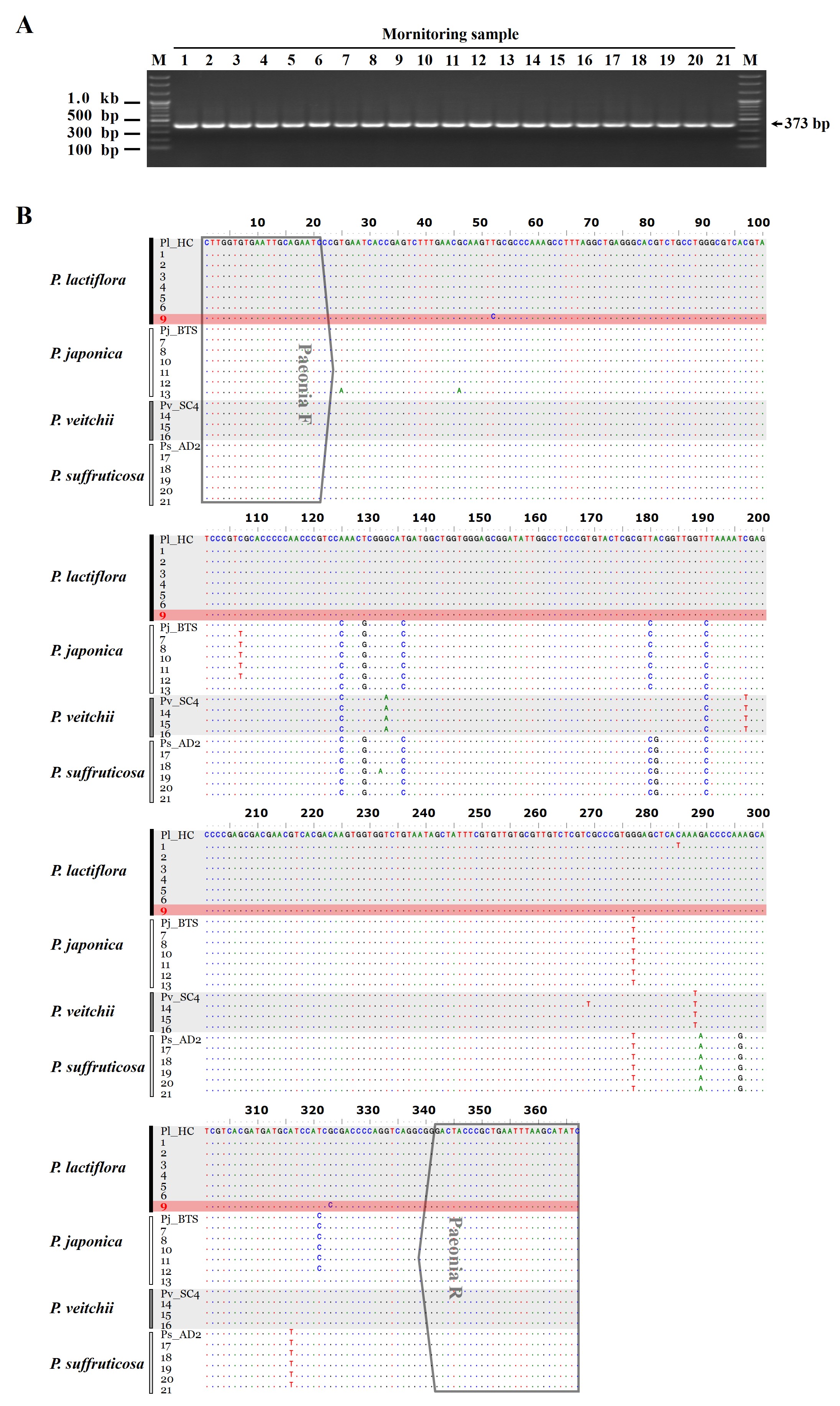

Supplement: Supplementary file 1 [file molecules-22-01922-s001.zip › Supplemental Figure 3..jpg]

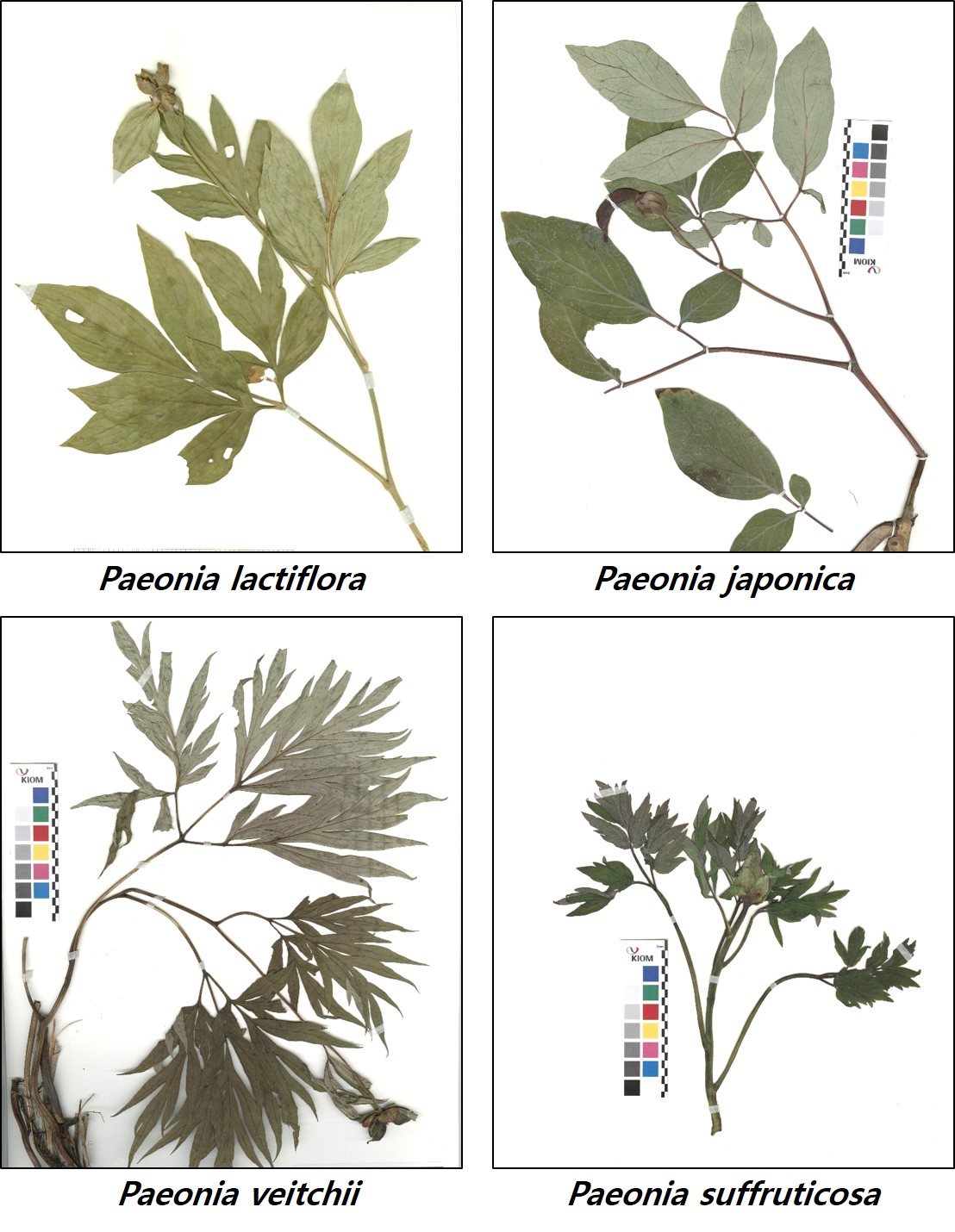

Supplement: Supplementary file 1 [file molecules-22-01922-s001.zip › Supplemental Photo 1..jpg]
